# Bacteremia due to *Gordonia polyisoprenivorans*: case report and review of literature

**DOI:** 10.1186/s12879-017-2523-5

**Published:** 2017-06-12

**Authors:** Xiurong Ding, Yanhua Yu, Ming Chen, Chen Wang, Yanfang Kang, Hongman Li, Jinli Lou

**Affiliations:** Department of Clinical Laboratory, Beijing Youan Hospital, Capital Medical University, Beijing, 100069 China

**Keywords:** *Gordonia polyisoprenivorans*, Bacteremia, Case report, AIDS

## Abstract

**Background:**

*Gordonia polyisoprenivorans* is a ubiquitous aerobic actinomycetes bacterium that rarely cause infections in humans. Here, we report a case of *G. polyisoprenivorans* catheter-related bacteremia in an AIDS patient.

**Case presentation:**

A 37-year-old man with a past medical history of AIDS-related lymphoma suffered bacteremia caused by a Gram-positive corynebacterium. The strain was identified as a Gordonia species by matrix-assisted laser desorption ionization–time of flight mass spectrometry and confirmed to *G. polyisoprenivorans* by 16S rRNA combined with *gyrB* gene sequencing analyses. The patient was treated with imipenem and had a good outcome.

**Conclusions:**

The findings from our case and previously reported cases indicate that malignant hematologic disease, immunosuppression, and indwelling catheter heighten the risk for *G. polyisoprenivorans* infection. Molecular methods should be employed for proper identification of *G. polyisoprenivorans* to the species level.

## Background

*Gordonia polyisoprenivorans* is a ubiquitous gram-positive coryneform bacterium belonging to the family Gordoniaceae in the order Actinomycetales [1]. It was first isolated in 1999 from stagnant water inside a deteriorated automobile tire [2]. *G. polyisoprenivorans* is well known for being the most effective rubber-degrading bacteria, but not as a common cause of bacteremia [3]. Here, we report a case of bacteremia caused by *G. polyisoprenivorans* in a patient with AIDS and review similar cases reported in the literature to summarize the important features of this disease.

## Case presentation

A 37-year-old man with a past medical history of AIDS-related lymphoma was admitted to Beijing Youan Hospital on 12 July 2016, for a relapse of diffuse large B cell lymphoma. The patient had been diagnosed with AIDS-related lymphoma 1 year earlier, and received eight courses of chemotherapy with rituximab, doxorubicin, vincristine, and etoposide (R-EPOCH). The patient recovered well and was discharged, with advice for regular follow-up. Three months ago, the results of a positron emission tomography-computed tomography scan suggested lymphoma recurrence, and the patient was transferred to our institution with an indwelling central venous catheter (CVC) catheter that had been inserted 3 months earlier for administration of chemotherapy. After 3 days of chemotherapy with etoposide, ifosfamide, and cisplatin, the results of clinical laboratory tests revealed myelosuppression, with white cell count of 0.11 × 10^9^/L, hemoglobin of 4.0 g/dL, and platelet count of 8 × 10^9^/L. His body temperature had been abnormal for 2 days, reaching 38.6 °C at the highest point, with procalcitonin of 0.96 μg/L, ESR of 56 mm/h, and CD^4+^ T-lymphocyte count of 52 × 10^6^ cells/L. A physical examination revealed no specific signs of infections or skin manifestations. Two sets of blood cultures, one from a peripheral vein and the other through the CVC (each 10 ml in volume), were collected and sent to the laboratory for examination. The patient was started on empirical antimicrobial therapy with imipenem.

The blood culture drawn through the CVC was first flagged as positive by a BACTEC™ FX instrument (Becton, Dickinson and Company, USA) after 38 h of incubation in an aerobic bottle. After 61 h of culture in an aerobic bottle, the peripheral blood culture also became positive. Direct microscopic examination based on Gram staining revealed the presence of nonsporulating beaded Gram-positive bacilli. Subcultured blood specimens were plated on sheep blood agar and MacConkey agar. On the sheep blood agar incubated at 35 °C in an aerobic environment with 5% CO_2_, small white colonies became evident within 24 h. After 3 days of incubation, the colonies became mucoid, and the colonies turned yellow-orange. The bacteria were positive for catalase, negative for cytochrome oxidase activity, nonmotile, and unable to grow anaerobically.

Bacterial identification was performed by matrix-assisted laser desorption ionization–time of flight mass spectrometry (MALDI-TOF MS) according to the manufacturer’s instructions, and the obtained protein profiles were processed and analyzed by MALDI Biotyper 3.0 software (Bruker Daltonics, Germany). However, the MALDI-TOF MS failed to confidently identify the isolate to the species level. Nevertheless, the isolate was identified as a *Gordonia* species, with a top match score of 1.764 (for *Gordonia rubripertincta*; scores of ≥2.0 and <2.0 to ≥1.7 represent identification to the species level and genus level, respectively), suggesting it did not resemble any known *Gordonia* species in the database.

Thereafter, bacterial DNA extraction, PCR amplification, and DNA sequencing of the 16S rRNA with a universal primer pair were conducted to confirm the results. The obtained product sequence (1404 bp) was compared with published sequences in the GenBank database (http://www.ncbi.nlm.nih.gov/blast). The results showed that the isolate had 99% matches with the type strains of *G. polyisoprenivorans* (strains W8130 and VH2), *Gordonia bronchialis* (strain DSM 43247), and *Gordonia terrae* (strain EY-T12). Sequencing of the *gyrB* genes was then performed according to a previous report [4]. The results showed that the *gyrB* gene of the isolate had 99.0% sequence identity with the gene sequence of the *G. polyisoprenivorans* strain, indicating that the isolate was *G. polyisoprenivorans*.

The isolate was sensitive to amikacin, ampicillin, amoxicillin-clavulanate, cefotaxime, imipenem, meropenem, ciprofloxacin, minocycline, linezolid, and vancomycin, with intermediate sensitivity to trimethoprim-sulfamethoxazole.

At 3 days after the start of imipenem therapy, there was a complete disappearance of fever and a remarkable improvement in the patient’s clinical status. From day 5 onward, the patient was switched to oral antibiotics. As there was no swelling or effusion around the CVC, the catheter was not removed. The patient was followed up for 3 months. There was no recurrence of the infection during the follow-up period. However, he died after 3 months apparently from progression of his hematological malignancies.

## Discussion and conclusions

In recent years, *Gordonia* species have increasingly been recognized as pathogens that cause human infections in both immunocompromised and immunocompetent individuals [5–7]. The major pathogenic *Gordonia* species are *Gordonia sputi*, *G. bronchialis*, and *G. terrae* [7–10]. Infection caused by *G. polyisoprenivorans* is very rare. Our review of the literature revealed only five cases of infections caused by *G. polyisoprenivorans* in patients aged 17–78 years that were described in detail [1, 11–14]. The clinical spectra of these cases, as well as that of our case, are summarized in Table 1. In all cases, the patients presented with systemic bacteremia and suffered from hematologic malignancies. Three of the five bacteremia cases were caused by a contaminated CVC, and the other two cases had histories of indwelling medical devices. It was reported that *G. polyisoprenivorans* can use isoprene rubber as its sole source of carbon and energy, and can form biofilms on intravascular devices. Notably, *Gordonia* species have been most commonly reported as causes of indwelling device-associated infections [6, 14, 15]. Our patient’s history of AIDS, hematologic malignancy, chemotherapy, and indwelling intravascular catheter may have predisposed him to this rare infection. Thus, all the aforementioned cases had hematologic malignancies and had undergone medical device implantation operations, which are generally known as major risk factors for bloodstream infections caused by *G. polyisoprenivorans*.

**Table 1:** Bloodstream infections of humans with *Gordonia polyisoprenivorans* and underlying conditions

Case	Authors	Age/Gender	Yr of isolation /Region	Type of infection	Identification method	Underlying conditions	Treatment
1	Verma P et al. [11]	78/M	2003/USA	Endocarditis	16S rRNA gene sequence and DNA-DNA hybridization	HHT,MDS, pancytopenia	imipenem and amikacin
2	Kempf VA et al. [12]	24/F	2004/Germany	Bacteremia due to CVC	16S rRNA gene sequence	CML, s/p-BMT	piperacillin-tazobactam
3	Langer AJ et al. [13]	17/ F	2007/USA	Bacteremia due to CVCand pneumonia	16S rRNA gene sequence	AML	vancomycin and ceftazidime; ciprofloxacin and azithromycin
4	Gupta M et al. [1]	17/F	2008/USA	pneumonia with associated bacteremia	16S rRNA gene sequence	AML	ciprofloxacin, zithromycin, vancomycin, ceftazidime
5	Ramanan P et al. [14]	48/M	2012/USA	Bacteremia due to CVC	16S rRNA gene sequence	AML, s/p-allogeneic PBSCT	ceftriaxone
6	present	37/M	2016/China	Bacteremia due to CVC	16S rRNA and gyrB gene sequence	AIDS-related lymphoma	imipenem

*F* female, *M* male, *HHT* hereditary hemorrhagic telangiectasia, *MDS* myelodysplastic syndrome, *CML* chronic myelogenous leukemia, *BMT* bone marrow transplant, *s/p* status post, *AML* acute myelogenous leukemia, *PBSCT* peripheral blood stem cell transplant, *AIDS* Acquired Immune Deficiency Syndrome

Using conventional microbiologic culture and biochemical analyses, *Gordonia* species are particularly difficult to identify to both the genus and species levels. They are sometimes dismissed as *Corynebacteria*, *Nocardia*, or *Rhodococcus* species [12, 13, 16, 17]. It is also believed that the incidence of *Gordonia* infections has been underestimated. In our review of the literature, all of the *G. polyisoprenivorans* isolates were confirmed to the species level by 16S rRNA gene sequencing analyses, and sometimes also required combination with *secA1* or *gyrB* gene fragment sequencing. This could be one reason why there have been few reports on *G. polyisoprenivorans* infections in the clinic. In the present case, although MALDI-TOF MS failed to identify the isolate to the species level (*G. polyisoprenivorans* is not in the Biotyper 3.0 database), it did identify the isolate as a *Gordonia* species. Moreover, Lam et al. [17] reported that *G. sputi* and *G. bronchialis* can be confidently identified to the species level by MALDI-TOF MS. Therefore, we believe that with the continuous improvement and development of the database, MALDI-TOF MS will become a powerful tool for identifying *Gordonia* species, and that increasing numbers of infections caused by *Gordonia* species will be found in the clinic.

There are no standardized recommendations for the treatment of infections caused by *Gordonia spp.* The available data suggest that *Gordonia spp.* are generally susceptible to many antimicrobial drugs [5, 6] In a report on the antimicrobial susceptibility of 13 clinical isolates and one type strain, Moser et al. [18] found that *G. polyisoprenivorans* had high susceptibility to amikacin, ampicillin, ceftriaxone, imipenem, amoxicillin-clavulanate, ciprofloxacin, vancomycin, and linezolid, but poor susceptibility to trimethoprim-sulfamethoxazole, clarithromycin, tigecycline, and minocycline, with 36–57% of tested isolates showing higher minimal inhibitory concentrations. The isolate in the report by Langer et al. [13] also showed resistance to trimethoprim-sulfamethoxazole. In the present case, we also found that *G. polyisoprenivorans* was insensitive to trimethoprim-sulfamethoxazole. However, in patients with AIDS, trimethoprim-sulfamethoxazole has never been prophylactically used to reduce the opportunity of infection. Therefore, the possibility for initial resistance of *G. polyisoprenivorans* to trimethoprim-sulfamethoxazole needs to be validated by a larger study in a clinic. It is also worth noting that *G. polyisoprenivorans* has strong biofilm formation ability. It has been clearly validated that sessile bacterial communities in biofilms exhibit decreased susceptibility to antibiotics compared with planktonic organisms, and that conventional susceptibility methods cannot accurately predict the actual susceptibility of organisms existing in a biofilm [19]. Thus, to prevent persistent infections, the resistance of *G. polyisoprenivorans* should be considered when selecting an appropriate treatment method. Under certain conditions, catheter removal may be the best approach.

In conclusion, *G. polyisoprenivorans* is a rare but emerging human pathogen that causes bloodstream infections in immunocompromised hosts. Malignant hematologic disease, immunosuppression, and indwelling catheter heighten the risk for *G. polyisoprenivorans* infection. As these bacteria are difficult to identify to the species level in routine laboratories, 16S rRNA gene sequencing techniques and MALDI-TOF MS are recommended for accurate diagnosis. Treatment of a *G. polyisoprenivorans* infection should be based on the results of in vitro susceptibility tests.

## Abbreviations

CVC: Central venous catheter
MALDI-TOF: Matrix-assisted laser desorption ionization–time of flight mass spectrometry
